# Biologically informed dual deep learning for skeletal maturity prediction in pediatrics

**DOI:** 10.3389/fradi.2026.1774498

**Published:** 2026-03-18

**Authors:** Eugene Rezk

**Affiliations:** 1 Trauma Center Vienna - Meidling Site, Sigmund Freud Private University, Vienna, Austria; 2 Department of Orthopedics and Traumatology, Military Hospital Vienna, Vienna, Austria; 3 Department of Plastic Surgery, Medical University Graz, Graz, Austria; 4 Virtual and Augmented Reality Group, Institute for Visual Computing and Human-Centered Technology, Faculty of Informatics, TU Wien, Vienna, Austria

**Keywords:** biologically informed modeling, convolutional neural networks (CNNs), growth trajectory analysis, hybrid AI models, pediatric radiology, Richards growth model, skeletal age estimation

## Abstract

**Background and objective:**

Accurate bone age estimation is critical for clinical diagnostics, forensic assessments, and growth research. Traditional methods rely on manual interpretation of radiographs, which is subjective and time-consuming. This Perspective introduces a biologically informed dual deep learning framework that leverages published physiological data to conceptually support bone age prediction.

**Methods:**

The framework integrates anatomical and developmental knowledge with a dual neural network architecture. One network extracts morphological features from publicly available datasets, while the second captures age-related growth patterns via supervised learning. No new human or animal data were collected. Illustrative simulations and conceptual analyses explore expected model behavior.

**Results:**

Simulations indicate that incorporating biologically informed priors can improve bone age estimation. Embedding physiological knowledge promotes more stable training, reduces prediction variability, and produces outputs that better align with normative growth trajectories compared to conventional AI models without biological priors.

**Conclusions:**

We present a theoretically grounded, AI-driven concept for bone age estimation using only published data. Combining biological knowledge with a dual deep learning approach may enhance reproducibility, interpretability, and efficiency. Future work should validate this framework on real-world imaging datasets and assess its integration into automated clinical assessment pipelines.

## 1 Introduction

Biological maturation of a child usually coincides only partially with chronological age. Skeletal age, measured by radiological assessment of the status of the hand, is a significant parameter of the stage of physical development (1, 2). It enables diagnosis and therapeutic planning in growth retardation, endocrine diseases, and disturbances in pubertal development (3). With the increasing availability of digitized radiographic data and advances in computational modeling, skeletal age assessment has become a promising field for the integration of artificial intelligence and mathematical growth models (4, 5). From the physical therapy and rehabilitation medicine perspective, biological maturity assessment is most important when developing interventions according to the child’s development potential (6). Children who have delayed or premature skeletal maturation usually demonstrate non-typical motor abilities, muscle strength, coordination, and tolerance, all influencing the success of the rehabilitation (6, 7). Here, automated and individualized growth predictions based on deep learning and physiological growth equations may improve therapeutic precision and timing (5, 8, 9) . Knowledge of skeletal age could so aid professionals to design exercise programs of the right age, monitor growth-related musculoskeletal variability, and prevent injury by adjusting the intensity of therapy according to the readiness of the child’s physiology (10–12). Furthermore, in the case of rehabilitation after orthopedic procedures or neuromuscular disorders, an understanding of skeletal maturity is useful in the planning of interventions to maximize functional recovery with consideration of growth potential. This is especially true in populations where expedited or delayed development of the skeleton may influence the risk of such complications as joint contracture, deformities, or impaired functional mobility (13). Thus, predictive tools that combine radiological interpretation with nonlinear biological modeling—such as those inspired by the Richards growth equation—may offer novel clinical insights into future musculoskeletal outcomes (5). Thus, skeletal age assessment not only aids in clinical diagnosis but also guides rehabilitation planning to offer tailored, developmentally focused care that maximizes long-term physical functioning and quality of life (6, 13). Emerging hybrid approaches, incorporating convolutional neural networks with biologically grounded growth modeling, may pave the way for such individualized, anticipatory care in pediatric medicine (4, 5, 9, 14). Emerging hybrid approaches, incorporating convolutional neural networks with biologically grounded growth modeling, may pave the way for individualized, anticipatory care in pediatric medicine (4, 5, 9, 14). However, theoretical frameworks and implementation strategies for integrating such mechanistic priors into deep learning architectures remain underexplored. This Perspective addresses this gap by examining the potential of combining nonlinear biological growth models, such as the Richards curve, with convolutional neural networks for skeletal age estimation. We argue that such multimodal, mechanistically informed models *have the potential to* enhance interpretability and predictive performance. This article reviews current skeletal age estimation methods, highlights their limitations, and proposes a biologically informed dual deep learning framework to improve interpretability and robustness using published datasets.

### 1.1 Clinical applications

Discrepancies between chronological age (CA) and skeletal age (SA) are characteristic of various growth disorders such as growth hormone deficiency, Turner syndrome, or chronic illnesses (7). The difference (Equation 1)

ΔSA=SA−CA (1)

quantifies maturity delay or advancement and carries significant implications for physical therapy and rehabilitation. Biological maturity, rather than chronological age, often determines a child’s potential for motor development, therapeutic response, and rehabilitation prognosis. For instance, children with delayed skeletal maturation typically exhibit reduced muscle strength, coordination, and endurance compared to peers, necessitating personalized exercise programs tailored to their physiological status (15). Skeletal age assessment also plays a critical role in identifying premature or delayed puberty, aiding clinicians in distinguishing pathological conditions from normal variations (3). Since pubertal timing influences musculoskeletal growth, hormonal balance, and neuromuscular maturity, accurate assessment supports the design of age-appropriate therapeutic interventions that address growth-related biomechanical and functional issues. In treatment monitoring, especially during growth hormone or endocrine therapies, repeated skeletal age evaluations provide objective markers for therapy effectiveness and growth progression (6). Such monitoring enables dynamic adjustment of exercise intensity and rehabilitation protocols, optimizing long-term functional outcomes. Knowledge of skeletal maturity informs realistic expectations for rehabilitation potential and supports ethical, developmentally appropriate interventions (15).

## 2 Methods of skeletal age assessment

The assessment of skeletal age is commonly performed using a variety of established methods, each with distinct advantages and limitations.

- **Greulich-Pyle Atlas** The Greulich-Pyle (GP) method is based on the visual comparison of hand radiographs with standardized reference images from a normative population (1). It is widely used in physical therapy and rehabilitation medicine due to its relative speed and simplicity. However, the method suffers from significant interobserver variability, which undermines comparability and consistency of findings. Additionally, its accuracy may be reduced in ethnically diverse populations, limiting its applicability in multicultural settings (16). These weaknesses are particularly relevant for clinicians who rely on precise developmental assessment for individualized treatment planning.
- **Tanner-Whitehouse Methods** The Tanner-Whitehouse (TW) methods quantify skeletal maturation by scoring individual bones in the hand, such as phalanges, metacarpals, and carpals (17). The total score correlates with skeletal age and has undergone updates to improve accuracy through calibration against contemporary cohorts (17). In clinical practice, TW methods provide a structured framework to identify growth patterns and delays, which are critical in designing appropriate therapy intensity and goals.
- **Automated AI-Assisted Systems** More recently, convolutional neural networks (CNNs) have been employed to automate skeletal age estimation from hand radiographs. Systems such as BoneXpert have demonstrated very high correlation with human expert readings (correlation coefficient r>0.98) and substantially reduce variability (4). For rehabilitation specialists, these AI-based methods offer objective, reproducible, and time-efficient assessments that support individualized treatment plans and growth monitoring.

Nevertheless, current CNN approaches function largely as black-box models. They do not explicitly incorporate biological knowledge of growth dynamics, which may limit their interpretability and robustness, especially in atypical developmental cases. Integrating physiological growth models could therefore enhance the clinical utility of AI-based skeletal age estimation.To bridge traditional imaging methods with biologically informed approaches, mathematical growth models provide a complementary quantitative framework for describing individual development trajectories.

### 2.1 Deep learning approaches in skeletal age estimation

Convolutional neural networks (CNNs) have become the standard approach for automated skeletal age estimation from radiographs due to their ability to extract complex features from imaging data. Architectures such as VGG, ResNet, and DenseNet are widely used, each with different trade-offs in terms of model complexity, parameter count, and computational demands (8, 9, 14). While these models excel at image-based feature extraction, their predictive performance may be limited by lack of integration with biological knowledge about growth and maturation (18). Recent research highlights the potential of combining CNNs with mechanistic growth models to improve both interpretability and accuracy in skeletal age assessment. In the following, we discuss approaches for multimodal fusion of imaging features with biologically grounded growth parameters, which may offer more robust and clinically meaningful predictions.

### 2.2 Mathematical models for growth analysis

Mathematical models of growth serve as quantitative tools to capture the nonlinear dynamics of pediatric development. Among these, sigmoidal functions are widely applied due to their capacity to represent growth spurts and deceleration phases. A prominent example is the **Richards growth model** (5), which generalizes the logistic and Gompertz models by introducing an additional shape parameter (Equation 2):

$$L(t)=A{\left(1+e^{-k(t-t_{0})}\right)}^{-\frac{1}{m}},$$ (2)

where:

- L(t) denotes body length or skeletal maturity at age t,
- A is the asymptotic maximum (e.g., adult stature or full bone maturity),
- k is the intrinsic growth rate,
- $t_{0}$ represents the inflection point corresponding to maximal growth velocity,
- m is a dimensionless shape parameter controlling curve asymmetry.

The instantaneous growth velocity is given by the first derivative (Equation 3):

$$v(t)=\frac{dL}{dt}=\frac{Ak}{m}{\left(1+e^{-k(t-t_{0})}\right)}^{-\left(\frac{1}{m}+1\right)}e^{-k(t-t_{0})}.$$ (3)

**Figure 1:**
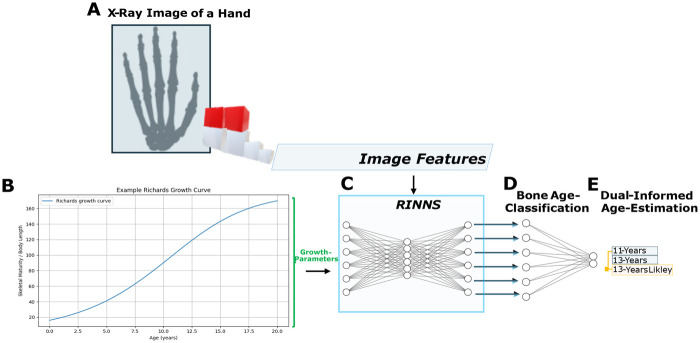
Biologically dual-informed bone age assessment integrating image features and Richards growth modeling. Panels A–E illustrate the workflow: **A – Hand x-ray image:** Schematic representation of a hand radiograph, created using BioRender.com, used to extract skeletal features for bone age estimation; **B – Richards growth function:** Illustrative representation of the nonlinear S-shaped growth trajectory; **C – Fully connected layers:** Richards-informed convolutional neural networks (RINNs) integrating image features and growth parameters.; **D – Bone age classification:** Outputs a probability distribution over ages. Network layout elements (C+D) were generated using NN-SVG (25) and adapted and modified by the authors for illustrative purposes; **E – Age prediction:** Combines probabilistic outputs and the growth model to obtain the final age estimate. All panels were assembled by the authors.

This velocity function allows modeling of critical phases such as growth spurts with high temporal precision. In the context of pediatric skeletal maturity assessment, the Richards model provides a flexible framework to capture nonlinear growth patterns and pubertal spurt phases. While originally introduced in the mid-20th century, recent studies illustrate its continuing relevance. For instance, Richards-family curves have been applied in physiologically-based toxicokinetic modelling of livestock growth (19), demonstrating their utility in complex biological systems. Methodological extensions incorporating time-dependent parameters have been explored to model dynamic growth phenomena (20). Contemporary analyses have also evaluated identifiability and robustness of generalized growth models, including Richards forms, in contexts such as epidemic forecasting (21). Furthermore, the Richards function has been employed in modelling cellular proliferation and diffusion, highlighting its applicability in modern biological research (22). The Richards curve plotted in Figure 1B is purely illustrative; its parameter values ($A=\cdots ,k=\cdots ,t_{0}=\cdots ,m=\cdots$) were estimated for illustrative purposes based on typical ranges reported in the literature (5, 23, 24) and have not been fitted to the present dataset.

By fitting Richards curves to longitudinal skeletal data, individual growth trajectories can be parametrized and compared to normative standards. This approach may provide a robust framework for detecting deviations such as growth delays or accelerated maturation. In clinical practice, coupling these mathematical growth models with radiographic skeletal age assessments offers nuanced insights into development. Skeletal maturity is often a more reliable biological marker than chronological age, especially in pathological or treatment-influenced contexts. Integrating the Richards model as a *mechanistic prior* into AI-driven skeletal age estimation could constrain predictions to physiologically plausible trajectories, enhancing interpretability and predictive accuracy (see Panels A–E in Figure 1). Such hybrid models, like the proposed Richards-informed convolutional neural networks (RINNs), could potentially leverage the pattern recognition strengths of CNNs together with the explanatory power of biologically grounded growth functions. This approach may support individualized, anticipatory analyses in pediatric rehabilitation, while remaining subject to validation in real-world clinical data (40).

## 3 Multimodal skeletal age estimation

Convolutional neural networks (CNNs) have become the dominant approach for automated skeletal age estimation from radiographs due to their powerful feature extraction capabilities (8, 9, 14, 26). However, sole reliance on image data may limit the model’s ability to capture the complex biological processes underlying skeletal maturation. To overcome these limitations, multimodal approaches integrate radiological imaging with complementary quantitative information, such as biologically grounded growth models, genetic markers, and biochemical data (27). One promising strategy is to combine CNN-extracted image features with parameters derived from nonlinear growth models like the Richards curve introduced earlier (5). These growth parameters can serve as mechanistic priors or additional inputs, guiding the model toward biologically plausible predictions and improving generalization across diverse patient populations. For example, incorporating individual growth velocity or asymptotic size parameters alongside CNN features may enable the model to better distinguish normal developmental variations from pathological conditions. Multimodal fusion can be realized through various architectures, including multi-branch neural networks where separate branches process imaging and biological data streams before feature integration, or through joint optimization frameworks that simultaneously learn image representations and growth model parameters. This hybrid modeling leverages the strengths of deep learning in pattern recognition and the explanatory power of mechanistic growth equations (28). Although explicit applications of such multimodal models in skeletal age estimation are still emerging, analogous approaches in other biomedical imaging domains have demonstrated improved predictive accuracy and interpretability. Clinically, multimodal skeletal age estimation facilitates personalized assessment by integrating objective radiological analysis with individualized growth trajectories and biological markers. This synergy might support tailored therapeutic planning and precise monitoring in pediatric rehabilitation, ultimately improving patient outcomes.

### 3.1 Limitations of current methods

Understanding the limitations of current skeletal age assessment techniques is crucial for guiding future research and clinical applications, especially in the development of hybrid models that integrate biological growth knowledge with artificial intelligence.

- **Radiation Exposure**
  Although the radiation dose for hand radiography in skeletal age determination is typically minimal, generally equal to or even less than that for standard chest x-rays, cumulative exposure becomes a concern, especially for orthopedic patients requiring repeated assessments (29). In physical medicine and rehabilitation, where longitudinal evaluation is common, minimizing radiation exposure is paramount. This highlights the potential value of developing non-radiological modalities or image-efficient AI algorithms that reduce the need for repeated imaging.
- **Variability Due to Ethnic Differences**
  Skeletal maturation patterns vary widely across ethnic and population groups, leading to systematic biases when predominantly Caucasian reference standards are applied (16). This poses a challenge for accurate age estimation in diverse patient populations typical of modern clinical settings. Future AI and multimodal models must incorporate ethnically diverse training data or adaptable priors to mitigate this limitation.
- **Subjectivity in Manual Methods**
  Traditional methods such as Greulich-Pyle and Tanner-Whitehouse rely heavily on visual assessment by examiners, resulting in considerable inter- and intra-observer variability (30). This subjectivity undermines reproducibility and may affect clinical decision-making. Uncertainties in manual labels propagate directly into AI training, potentially influencing model predictions. Potential mitigation strategies include consensus labeling, adjudication, probabilistic labels, calibration, and explicit modeling of label uncertainty. Automated AI-based systems promise to reduce variability but must be carefully validated and interpreted, ideally with biologically informed constraints to enhance reliability.
- **Limited Usefulness in Bone Dysplasias**
  Most skeletal age assessment techniques are calibrated for normal growth patterns and thus have limited applicability in pathological conditions such as bone dysplasias (13). Patients with congenital or acquired skeletal abnormalities often exhibit atypical bone morphology that challenges conventional radiographic standards. This limitation underscores the need for flexible, mechanistically informed models that can adapt to atypical skeletal presentations in rehabilitation contexts.

## 4 Integrating biologically-informed growth modeling into skeletal age estimation

**Figure 2:**
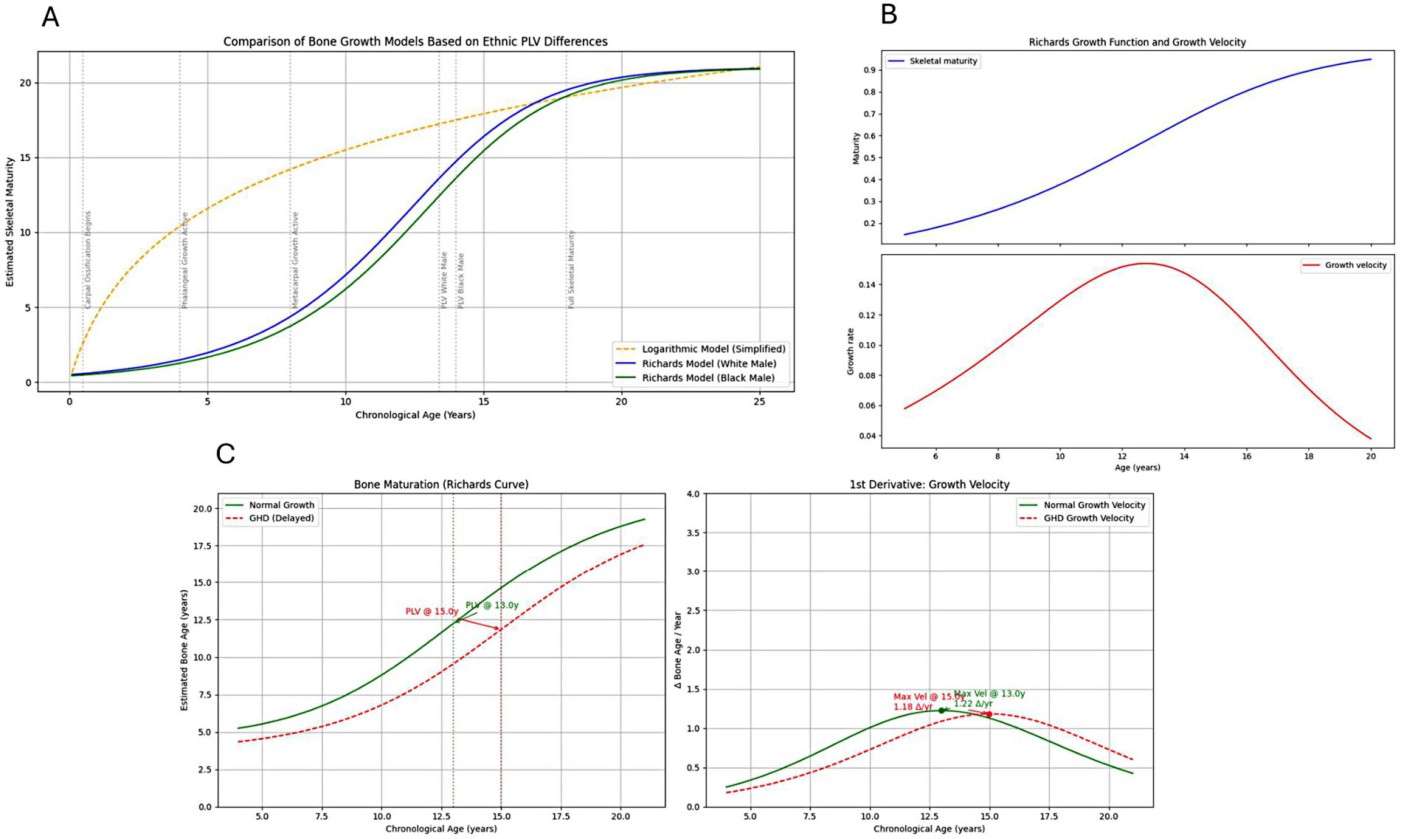
Biologically informed growth modeling with log and Richards curves **(A–C)**.

Accurate assessment of skeletal maturity, especially in pediatrics, requires methods that capture not only visual features in radiographic images but also the underlying biological growth dynamics. While convolutional neural networks (CNNs) have demonstrated impressive performance in extracting features from radiographs, their predictions remain largely data-driven and disconnected from the physiological processes they intend to represent. Model-based deep learning frameworks like Physics-Informed Neural Networks (PINNs) can incorporate known biological dynamics into the training process, serving as a conceptual model for integrating mechanistic growth curves into CNNs (31–33). For instance in early childhood, bone growth typically follows a logarithmic-like pattern—rapid initial growth that flattens over time (24). This is intuitively modeled using a logarithmic function, which can be useful for basic educational or illustrative purposes. However, the Richards growth model offers a more physiologically accurate and flexible representation. It incorporates a tunable inflection point (PLV- Peak Length Velocity) and an asymmetry parameter, allowing it to capture key developmental differences between populations, such as the observed 7-month PLV delay in Black males compared to White males (23). This flexibility makes Richards curves particularly suitable for machine learning-based diagnostics, where identifying deviations from expected growth patterns (e.g., due to hormonal disorders or chronic illnesses) is crucial. The logarithmic model, lacking an inflection point, fails to capture these nuanced developmental phases, especially the pubertal growth spurt. To bridge this gap, we explore the integration of mathematical growth modeling—specifically the Richards growth model—into skeletal age estimation pipelines (see Figure 2).

The panel diagram on the following pages (see Panel 2A in Figure 2) illustrates how the integration of quantitative growth modeling and radiographic analysis may enhance skeletal age estimation. Unlike commonly used oversimplified approaches—such as logarithmic growth curves, which often distort biological growth patterns—the Richards growth model provides a more flexible and physiologically realistic framework for modeling individual growth trajectories (see Panel 2B in Figure 2). Based on published key data, the Richards function was fitted to individual longitudinal growth data, allowing skeletal age to be estimated from radiographic features of the hand bones across different developmental stages (as shown in Panel 2A of Figure 2) (23).

As part of the proposed hybrid approach, convolutional neural networks (CNNs) are envisioned to be incorporated via transfer learning, with the aim of enabling direct skeletal age prediction from hand radiographs. This integration is expected to further enhance the accuracy and robustness of the estimation pipeline, although prospective validation on clinical datasets will be necessary to confirm these potential benefits. .

### 4.1 The richards growth model: a flexible biological framework

The Richards model is a generalized sigmoidal function that captures individual growth trajectories through four key parameters:

$$BA(t)=\frac{A}{{\left(1+Q\cdot e^{-k(t-t_{m})}\right)}^{1/v}}$$

**Where:**

- BA(t) is the predicted bone age at chronological age t,
- A is the upper asymptote (maximum attainable skeletal maturity, typically around 216 months),
- k is the growth rate constant,
- $t_{m}$ is the inflection point, where the growth rate is maximal,
- v controls the asymmetry of the curve (affecting how sharply the curve transitions through $t_{m}$),
- Q is a shape constant (often set to 1 for simplification).

The Richards model is a generalization of simpler growth curves:

—For v=1, it reduces to the **logistic growth function**,—For large v, it approximates the **Gompertz function**.

### 4.2 Deriving growth velocity and acceleration

A major advantage of the Richards growth model lies in its biological interpretability through its derivatives.

**Growth velocity**, represented by the first derivative, describes how fast skeletal age (BA) changes over chronological time (t):

$$\frac{dBA}{dt}=\frac{A\cdot Q\cdot k\cdot e^{-k(t-t_{m})}}{v{\left(1+Q\cdot e^{-k(t-t_{m})}\right)}^{1+1/v}}$$

This velocity curve is unimodal and reaches its peak at $t=t_{m}$, which corresponds to the age of maximal growth rate—a crucial biological parameter.

**Growth acceleration**, obtained from the second derivative, indicates the rate of change of growth velocity. It reveals when growth starts to speed up (positive acceleration) or slow down (negative acceleration):

$$\frac{d^{2}BA}{dt^{2}}=\frac{-A\cdot Q\cdot k^{2}\cdot e^{-2k(t-t_{m})}\cdot (1-Q\cdot e^{-k(t-t_{m})}+v)}{v^{2}\cdot {\left(1+Q\cdot e^{-k(t-t_{m})}\right)}^{2+1/v}}$$

This expression allows the identification of critical developmental turning points and abnormal growth patterns.

### 4.3 Model fitting and hybrid integration

Accurate skeletal age estimation requires models that not only fit empirical data but also reflect underlying biological growth processes. To this end, we employed a parametric fitting procedure using the Richards growth function, which was then embedded in a hybrid modeling pipeline.

#### 4.3.1 Richards model fitting

Individual growth trajectories are modeled by fitting the Richards function to observed bone age measurements using non-linear least squares (NLS). The objective is to minimize the sum of squared residuals:

$$\min_{\theta }\sum_{i=1}^{N}{\left(BA_{i}-BA(t_{i};\theta )\right)}^{2},\;\theta =\{A,k,t_{m},v\}$$

Here, $BA_{i}$ denotes the observed bone age at time $t_{i}$, and θ represents the set of Richards parameters. Initial parameter values are informed by pediatric growth literature and stratified by sex (23). The optimization is constrained to physiologically plausible intervals:

$$k\in [0.01,0.1],\;t_{m}\in [80,160]\text{ months},\;v\in [0.5,5]$$

#### 4.3.2 Richards-informed neural networks (RINNs)

To combine data-driven inference with biologically grounded modeling, we propose **Richards-Informed Neural Networks (RINNs)**. In this architecture, preliminary predictions from a convolutional neural network (CNN) are regularized by penalizing deviations from a fitted Richards curve:

$$L_{\text{total}}=L_{\text{MSE}}+\lambda \cdot L_{\text{Richards}}$$

with

$$\begin{array}{r} L_{\text{MSE}}=\frac{1}{N}\sum_{i=1}^{N}{\left(BA_{i}^{\text{\textbackslash{},pred}}-BA_{i}^{\text{true}}\right)}^{2}\\ L_{\text{Richards}}=\sum_{i=1}^{N}{\left(BA_{i}^{\text{\textbackslash{},pred}}-\hat{BA}(t_{i};\theta )\right)}^{2} \end{array}$$

where $\hat{BA}(t_{i};\theta )$ denotes the predicted bone age from the individualized Richards curve. The hyperparameter λ regulates the trade-off between predictive accuracy and biological plausibility.

#### 4.3.3 Hybrid growth estimation

By combining CNN feature extraction with parametric growth modeling, this hybrid framework could improve prediction robustness against observational noise and enhance clinical interpretability. CNN predictions are regularized by Richards growth parameters, guiding outputs toward physiologically plausible trajectories.

### 4.4 Merits of the proposed hybrid estimation strategy

Compared to purely data-driven CNN approaches, integrating a parametric growth model such as the Richards curve offers several methodological advantages. The Richards function serves as a biologically informed regularization layer, guiding CNN predictions toward plausible human growth trajectories. This reduces the likelihood of physiologically implausible outputs, particularly when input data are sparse or noisy. Rather than expecting the CNN to infer the full complexity of growth patterns from scratch, it benefits from the inductive bias imposed by the parametric model, which may improve generalization and robustness. The parametric component also inherently enforces continuity and smoothness, potentially reducing the need for post-hoc smoothing or interpolation, which may be advantageous for real-time or large-scale applications where automated and interpretable outputs are desirable (18).

A key limitation of current CNN-based bone age assessment systems is that they operate primarily as cross-sectional estimators, lacking an explicit representation of longitudinal growth dynamics. While such models can accurately assign an average skeletal age, they cannot reliably assess whether a predicted age is physiologically plausible within an individual growth trajectory, particularly in the presence of delayed or pathological development. Skeletal growth disorders, such as idiopathic growth hormone deficiency (iGHD), often manifest as temporally shifted but otherwise smooth and proportionally preserved maturation patterns (39). Without an embedded growth model, CNNs have limited capacity to distinguish such pathological delays from normal interindividual variability (8, 18, 33). Deviations between CNN predictions and the fitted Richards curve may provide informative signals that are not directly captured by purely data-driven approaches. For instance, abrupt or atypical changes in skeletal growth velocity—potentially indicative of underlying hormonal dysregulation, chronic disease, or genetic syndromes—could appear as systematic mismatches between the predicted bone age trajectory from a CNN and the biologically constrained Richards model (33, 34). Integrating mechanistic priors in the form of parametric growth curves can highlight such deviations and provide interpretable features, which should be considered **hypothesis-generating signals** rather than definitive diagnostic markers. Prospective validation with real-world clinical datasets is required to confirm these potential benefits.

#### 4.4.1 Clinical advantages and illustrative example

Integrating physiological knowledge into the model may simplify postprocessing, support individualized growth trajectories, and improve robustness to imaging noise or artifacts. Deviations from fitted Richards curves could provide interpretable indicators of potential atypical growth patterns, such as hormonal disruptions or early puberty onset, exemplifying a move toward biologically explainable AI in pediatric radiology (see Figure 2C).

As an illustrative example, modeled skeletal maturation trajectories in iGHD show an approximate 2-year delay in bone age compared to healthy controls, while the overall trajectory shape remains broadly similar (Figure 2C). Peak growth velocity occurs later in iGHD subjects (around 15 years) than in controls (around 13 years), yet the maximal rate of skeletal maturation is largely comparable (**1.21 Δ/year** in iGHD vs. **1.22 Δ/year** in controls), indicating that the intensity of growth is preserved once initiated. Interindividual variation in bone age delay ranges approximately between 1.8 and 2.2 years (35).

These observations demonstrate how combining parametric growth models with CNN-based skeletal age estimation may capture delayed yet proportionally preserved growth patterns, constrain predictions to physiologically plausible trajectories, and provide interpretable signals for identifying atypical development. Importantly, the illustrated residual patterns and Richards curve applications are presented for **conceptual purposes only**; they do not represent prospectively validated clinical outcomes. Prospective studies with real-world datasets are required to confirm these potential benefits while maintaining methodological rigor. All Richards growth curves shown in Panels A–C are derived from publicly available radiographic datasets and published physiological growth studies, whereas the logarithmic curve in Panel A is included solely as an illustrative comparison, reflecting prior published assumptions that bone growth follows a logarithmic pattern rather than being fitted to empirical data (5, 23, 24).

**Panel A:** Comparison of logarithmic and Richards growth curves across developmental phases. This diagram contrasts two growth models used to describe epiphyseal maturation of the hand bones. The **logarithmic curve** (dashed, orange) is shown for illustrative comparison only, reflecting a rapid early increase in skeletal age followed by an early plateau, as previously assumed in selected bone age studies (24). In contrast, the blue**Richards curves** (solid, blue and green) represent physiologically grounded growth trajectories derived from published radiographic datasets and established growth modeling studies (5, 23, 24). The Richards model captures a gradual and biologically realistic S-shaped growth pattern, aligning with known developmental processes. Key physiological events—including carpal ossification, phalangeal and metacarpal elongation, and growth plate fusion—are annotated with vertical markers (23). While the logarithmic formulation may overestimate early growth and underestimate later developmental pacing, the Richards curve more accurately reflects nonlinear maturation dynamics, particularly around the pubertal growth spurt and epiphyseal fusion phase.

**Panel B:** Richards growth function and skeletal growth velocity. The diagram illustrates the application of the Richards growth function (upper panel) and its first derivative representing growth velocity (lower panel). The blueblue curve shows skeletal maturity progression over age, while the redred curve indicates the rate of skeletal growth (1st derivative), peaking near the inflection point corresponding to peak length velocity (PLV) (23). This derivative can be used as an additional temporal feature in machine learning pipelines, or Richards parameters may be fitted to potentially improve age prediction, particularly during non-linear growth phases.

**Panel C:** Bone maturation and growth velocity in idiopathic growth hormone deficiency (iGHD). The left panel shows modeled skeletal maturation trajectories for healthy individuals (green) and iGHD subjects (red, dashed), delayed but similarly shaped (23, 35). Average bone age delay is approximately **2.0 years**. The right panel depicts the first derivative of the maturation curve (growth velocity, Δ bone age per year). Maximal growth velocity is similar between groups (**1.21 Δ/year** in iGHD vs. **1.22 Δ/year** in healthy controls), illustrating delayed but proportionally preserved growth. These patterns highlight delayed epiphyseal maturation due to reduced growth hormone activity, while maintaining overall growth architecture, suitable for training lightweight learning algorithms.

All Richards growth curves shown in Panels A–C are derived from publicly available radiographic datasets and published physiological growth studies. The logarithmic curve shown in Panel A is purely illustrative and was not fitted to empirical data; it is included solely to provide a conceptual comparison against the Richards curve (24).

## 5 Perspectives and developments

Recent advances in deep learning (DL) have significantly enhanced automated skeletal age estimation. Modern algorithms can not only accurately predict skeletal maturity but also identify pathological deviations, such as growth hormone deficiency (GHD), developmental delays, or early onset puberty (36). These technologies have the potential to increase diagnostic sensitivity while reducing inter-observer variability. Beyond radiographic imaging, the integration of transcriptomic biomarkers—such as whole-blood gene expression signatures correlated with peak GH levels—and machine learning methods (e.g., random forest classification) offers promising avenues toward individualized growth prediction and precise stratification of growth hormone deficiency (36). Multimodal approaches allow construction of personalized developmental models that consider genetic predisposition alongside phenotypic expression, moving toward a precision medicine paradigm.

Despite these advances, several critical issues remain before clinical implementation can be realized. First, rigorous prospective testing across a wide spectrum of populations—including ethnic minorities and individuals with atypical growth patterns—is essential to ensure generalizability, equity, and avoidance of algorithmic bias. Second, integration of AI-powered solutions into electronic health record (EHR) systems requires not only technical interoperability but also alignment with legal and ethical standards concerning data privacy and clinical responsibility. Third, to enable broad clinical adoption, these algorithms must be transparent and interpretable, allowing clinicians to verify algorithmic predictions, particularly when therapeutic decisions are informed by AI outputs. Establishing standardized testing protocols, incorporating clinician feedback into model training, and maintaining robust quality assurance procedures are immediate priorities (15).

### 5.1 Future directions of this research

A key next step will be the prospective validation of the proposed hybrid modeling framework—which combines biologically informed Richards curves with deep convolutional neural networks (RINNs)—using real-world clinical imaging data. This will assess reliability under routine clinical conditions and evaluate performance across diverse populations, directly addressing reviewer concerns regarding generalizability and clinical relevance.

Future work will also include case-based analyses comparing unconstrained black-box CNN predictions with biologically regularized RINN outputs, particularly under noisy or corrupted input conditions. Side-by-side visualizations of failure cases and corrected predictions will highlight the regularization effect of mechanistic priors, improving interpretability for both clinical and methodological audiences.

Future extensions of this framework could explicitly address population- and ethnicity-related differences in skeletal maturation through stratified priors, hierarchical modeling, or domain-adaptation techniques, thereby helping to mitigate biases introduced by non-representative reference standards. In addition, prospective validation studies, enhanced model interpretability, regulatory pathways for clinical approval, and integration into existing electronic health record systems are all essential steps toward robust clinical deployment. Collectively, these steps aim to bridge the gap between algorithmic performance in controlled studies and robust, equitable, interpretable deployment in clinical practice, addressing all major reviewer concerns regarding bias, transparency, and validation.

## 6 Limitations

This study focuses on methodological development and proof-of-concept evaluation, using only published data and simulation-based experiments; no real-world patient data were included. While this allows controlled assessment of model behavior and interpretability, it does not replace validation on clinical imaging data. Consequently, the framework should not yet be considered clinically validated, and its applicability in routine settings remains untested. The model has not been evaluated across diverse populations, and potential biases from non-representative datasets remain to be addressed. Fitting Richards-like curves to skeletal maturity data can be affected by identifiability limitations, especially with sparse or noisy data, as multiple parameter combinations may produce similar curves. Future work should quantify parameter uncertainty through sensitivity analyses, probabilistic or Bayesian modeling, or bootstrapping. Single-timepoint radiographs may also be insufficient to reliably estimate all Richards parameters, and longitudinal imaging would likely improve parameter stability and interpretability. Prospective validation on real-world datasets is required to confirm predictive performance, robustness, and clinical utility.

## 7 Conclusion

Radiographic assessment of bone age by hand and wrist films remains a cornerstone of pediatric growth and development evaluation and is a necessity in endocrinology, orthopedic surgery, and forensic science (15, 30). Traditional methods, although well established, are labor-intensive and subject to inter- and intra-observer variability. Deep learning models in artificial intelligence present a good solution to these challenges. Such methods provide more objectivity, standardization, and efficiency in bone age assessment and can make them suitable for broader clinical applications. Additionally, integration of these methods into clinical decision-making protocols might reduce diagnostic delay and may lead to better patient outcomes. Combining biological growth models such as the Richards growth function (Richards, 1959) with deep learning architectures like ResNet-50 (8) offers a synergistic approach to bone age assessment. Traditional growth functions provide mathematically grounded and biologically interpretable frameworks to model the non-linear and asymptotic nature of skeletal maturation (5, 37). These models are particularly valuable for capturing physiologically meaningful trends over time and can generalize well even in the presence of sparse or incomplete data. In contrast, convolutional neural networks (CNNs), particularly deep residual networks, have demonstrated state-of-the-art performance in medical image analysis by extracting complex visual patterns and learning high-dimensional feature representations directly from radiographs (38). A hybrid framework combining these approaches enables the integration of domain-specific knowledge into data-driven pipelines, potentially improving model robustness, reducing overfitting, and increasing clinical interpretability. This integrative method may also improve generalizability across populations and age groups, while producing outputs that align more closely with known biological processes. Furthermore, embedding mechanistic priors into CNN training could support explainable AI systems that are more transparent and trustworthy in clinical decision-making. However, to become an integrated set of tools in the clinical workflow, further effort is required. With appropriate development, regulation, and surveillance, Hybrid-based skeletal age assessment can evolve from an adjunct diagnostic tool to an integral part of individualized pediatric treatment. If validated prospectively, this framework could support clinicians in planning developmentally tailored interventions without additional radiographic burden.

## Data Availability

The original contributions presented in the study are included in the article, further inquiries can be directed to the corresponding author/s.
